# The younger the better: importance of age in treatment of childhood obesity

**DOI:** 10.1007/s00431-023-05218-3

**Published:** 2023-09-27

**Authors:** Rasmus Møller Jørgensen, Amanda Bjørn, Vitus Bjørn, Jens Meldgaard Bruun

**Affiliations:** 1 Steno Diabetes Center Aarhus, Aarhus University Hospital, Palle Juul-Jensens Blvd 11, 8200 Aarhus, Denmark; 2 Institute of Clinical Medicine, University of Aarhus, Aarhus, Palle Juul-Jensens Blvd 11., Denmark; 3 Danish National Center for Obesity, Palle Juul-Jensens Blvd. 11, Aarhus, Denmark

**Keywords:** Obesity, Children, Lifestyle Intervention, Drop-out

## Abstract

Children living with obesity are prevalent worldwide. It is an established finding that many children who start a lifestyle intervention tend to leave prematurely. The aim of this study was to identify characteristics in children with obesity who prematurely leave a lifestyle intervention. The cohort study includes children living with obesity aged 4–17, treated in a Danish family-centered lifestyle intervention between 2014 and 2017. Data were collected from patient records. BMI-SDS was calculated using an external Danish reference population and multivariable regression analysis was used to answer the research question. Of the 159 children included, 64 children who left the intervention within the first 1.5 years were older compared to those who stayed in the intervention (10.2 years ± 2.9 vs 11.5 years ± 3.1, *p* = 0.005). Older participants (> 66.6^th^ percentile) had a shorter treatment duration (489 days) compared to the youngest (190 days 95% CI: 60; 320, *p* = 0.005) and middle third (224 days 95% CI: 89; 358, *p* = 0.001). Additionally, an inverse association was found between duration of treatment and age at baseline (−31 days, 95% CI (−50; −13), *p* = 0.001).

*Conclusion*: The risk of leaving a lifestyle intervention prematurely was primarily dependent on the age of the participants, emphasizing the importance of including children early in lifestyle interventions.

**Table:** 

**What is Known:** *•* *Lifestyle interventions for childhood obesity that are shorter in duration often lead to short-term weight reductions only. Limited knowledge exists on why some children prematurely leave these interventions.*	
**What is New:** *•* *This study observes a solid inverse correlation and association between age and time spent in the interventions, when treating childhood obesity. We hereby suggest age as an important determinant for the adherence to lifestyle interventions and emphasize the importance of treatment early in life.*

**What is Known:**

*•* *Lifestyle interventions for childhood obesity that are shorter in duration often lead to short-term weight reductions only. Limited knowledge exists on why some children prematurely leave these interventions.*

**What is New:**

*•* *This study observes a solid inverse correlation and association between age and time spent in the interventions, when treating childhood obesity. We hereby suggest age as an important determinant for the adherence to lifestyle interventions and emphasize the importance of treatment early in life.*

## Introduction

Childhood obesity is a rapidly growing global epidemic with a particularly high prevalence in developing countries [1, 2]. In Denmark, the prevalence of obesity increase with age. Approximately 1% of children aged 6–10 months, 3% of children aged 6–7 years, and 3–4% of children aged 14–15 years now living with obesity in Denmark [3]. Of the Danish adult population, 18.5% [4] are now living with obesity with an extra estimated cost of DKK 1.8 billion for treatment and care [5].

A social gradient of obesity has been observed in Danish children and adolescents [6], with the highest prevalence observed in families with lower socioeconomic status [6, 7], as defined by parental education and household income [6].

Childhood obesity is associated with an increased risk of developing non-communicable lifestyle diseases such as dyslipidaemia, hypertension, decreased insulin sensitivity (prediabetes), and hepatic steatosis [8–11]. In addition, children living with obesity often experience stigmatization, loneliness, and low self-esteem, compared to their peers [11], which can ultimately result in impaired quality of life and overt depression [10]. An association between obesity in childhood and obesity in adulthood has been established [12], which also increase the risk of developing diseases such as cardiovascular disease [13], type 2 diabetes [14], various types of cancers [15], and reduced life expectancy [16].

Lifestyle interventions are often the first step and a cornerstone in the treatment of childhood obesity [17, 18] with positive effects on both metabolic and psychological parameters [19, 20]. Furthermore, lifestyle interventions can result in weight reduction independent of age, socioeconomic class, and weight at initiation of treatment [21, 22]. Even though it may be argued that lifestyle interventions are the best approach to treat childhood obesity [23], the overall effect is still modest [24, 25], often due to low adherence. However, studies indicate that a long-term effect can be observed if the child adheres to a lifestyle intervention for at least ~ 1.5 years [23, 26].

Limited knowledge exists on why children and their families often prematurely leave a lifestyle intervention targeting childhood obesity [22, 27–31]. However, low socioeconomic status [29], lower parental educational level [30, 31], higher degree of obesity [27, 28, 31], and higher age of the participating child [26, 29] at treatment onset have all been reported to increase the risk of drop-out.

The objective of this study was therefore to identify characteristics in the child of the family which may increase the risk of prematurely leaving a lifestyle intervention targeting treatment of childhood obesity.

## Material and methods

### Study design

This retrospective longitudinal cohort study is a secondary analysis and contains data from a community-based intervention treating children living with obesity in the city of Randers, Denmark [23]. The intervention was originally initiated as a clinical treatment and not primarily as research. The study was therefore not randomized but did follow the CONSORT concept [23].

### The children’s obesity clinics treatment (TCOCT), Randers

As reported previously, the family-centered, multifactorial lifestyle intervention treating children and adolescents with obesity was a collaboration between the Department of Paediatrics at Randers Regional Hospital, Denmark, and specialized school nurses employed at four municipalities (Randers, Norddjurs, Syddjurs, and Faurskov) [23].

The participants were monitored with annual visits at the outpatient clinic at the hospital for up to 3 years (with a maximum of four visits). Between these visits, the children were treated by the specialized school nurses and were seen up to eight times per year.

At baseline, participants (i.e., children and their families) filled out questionnaires on predispositions (e.g., obesity, type 2 diabetes, cardiovascular disease, and mental health), previous weight loss attempts, parents marital status, and nationality. Anthropometric data (height and body weight), body composition, blood pressure, and blood samples, as well as quality of life, were collected during the yearly visit at the outpatient clinic. Body composition was assessed using a bio-impedance technique (Tanita BC-420MA).

### Subjects

As reported in Jorgensen et al. [23], a total of 199 children and adolescents with obesity (mean age 10.8 (± 3.1) and mean Body Mass Index Standard Deviation Score (BMI-SDS) 3.1 (± 0.3)) were included in the intervention between 1 January 2014 and 31 December 2017. The inclusion criteria for this study were obesity at baseline and participant age between 4 and 17 years. Obesity was defined as BMI-SDS above 2 SD (for age and sex) and BMI-SDS was calculated using validated Danish reference material [32]. For children leaving the intervention, the date and the reason for withdrawal were registered. The children were stratified into (1) completers or non-completers if adherence to the intervention was either longer or shorter than 1.5 years and (2) into age tertiles at baseline (i.e., 33.3^rd^ and 66.6^th^). Exclusion criteria were (1) no date for leaving the intervention and (2) children still enrolled in the interventions on 31 December 2017 and treated for less than 1.5 years. These children were excluded as it was uncertain if they would leave the intervention within the first 1.5 years.

The information on leaving the intervention was collected by the same nurse throughout the project. Either the parents were asked when they called the Department of Paediatrics to stop the treatment, or the nurse subsequently called the families. The date and the reason for leaving the intervention were then noted in the medical record.

### Data variables and statistical analysis

Data were collected from patients records at the Department of Paediatrics, Regional Hospital Randers, Denmark, and stored in REDcap [33], an electronic data capture tool.

To examine data for normally distribution, data were displayed in QQ plots and histograms. The QQ plots were then compared to other QQ plots of normally distributed data samples and same size.

Normally distributed data were reported as means with standard deviations (SD) and compared using a two-tailed Student *t*-test (two means) or one-way ANOVA (several means). Data found to be not normally distributed were reported as medians with interquartile range (IQR) and analyzed by using the Wilcoxon Rank Sum test (2 groups) or Kruskal-Wallis test (several means). The categorical variables were reported as *n* (%) and compared using Fisher’s exact test. Pearson’s correlation analysis was used to test for correlation between age and BMI-SDS at baseline and after the intervention.

Missing data in the baseline characteristics were kept as missing and not reported in the tables.

A multiple variable regression analysis was used to describe the association between “duration of the treatment” and (1) age at baseline and (2) BMI-SDS at baseline. The model was adjusted for sex, age, and BMI-SDS at baseline.

All estimates were reported with 95% confidence intervals (CI) and/or using 5% as the significance level. All analyses were done in Stata 17 College Station, TX: StataCorp LLC.

## Results

### Baseline characteristics

**Fig. 1:**
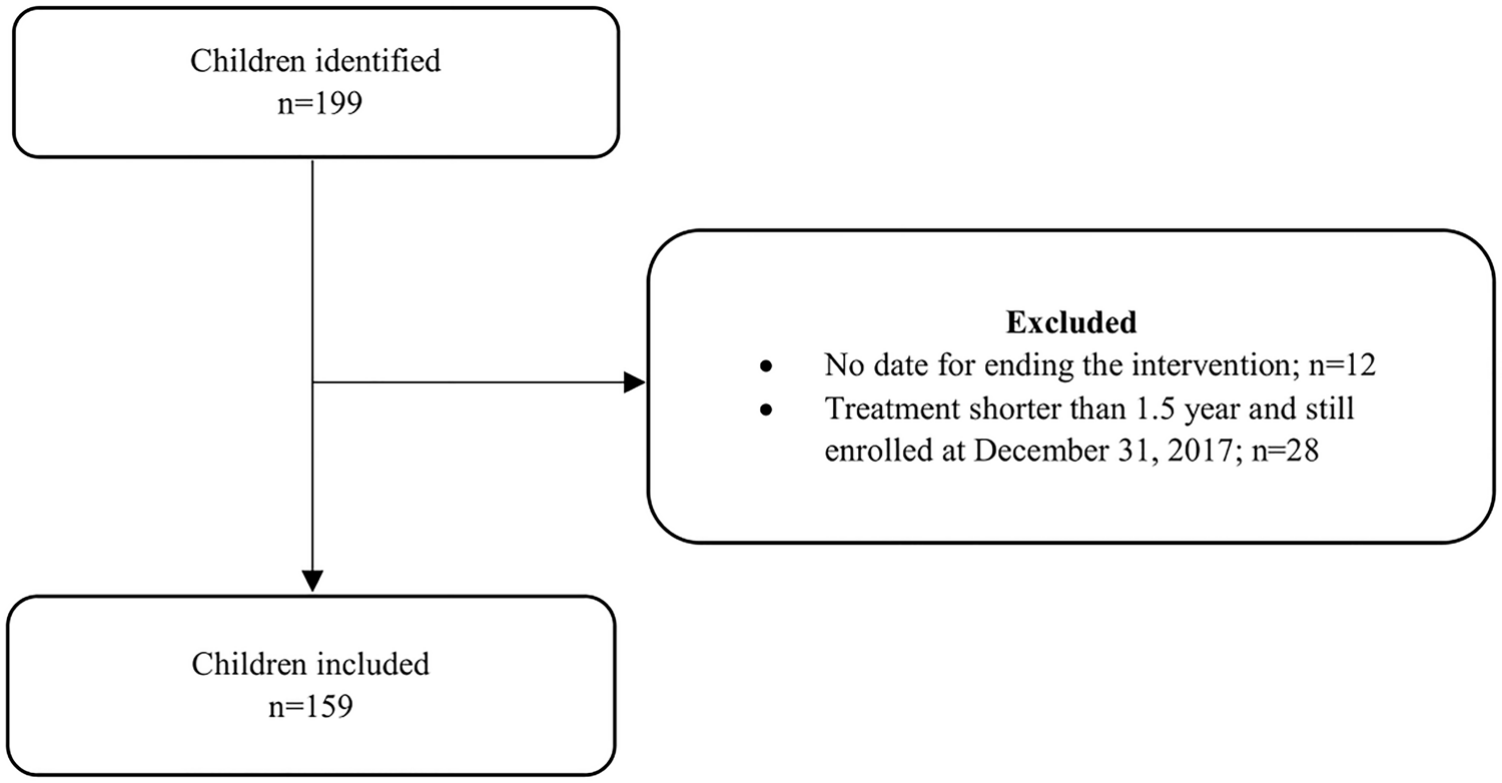
Flowcharts for inclusion and exclusion

Of the 199 children in the intervention, 159 children were included in this study and 31 were excluded: 12 children due to missing date for leaving the intervention, 28 due to still being enrolled on 31 December 2017 and treated less than 1.5 years (Fig. 1).

The 159 included children had a mean age of 10.7 (SD: ± 3.0) and a mean BMI-SDS of 3.1 (SD: ± 0.7). Of these, 142 children (90%) were predisposed to overweight and obesity, and 66 children (42%) reported previous weight loss attempts (Table 1).

**Table 1:** Baseline characteristics

Factor	Value
*N*	159
Sex - count (%)	
Boys	80 (50%)
Girls	79 (50%)
Age (years), mean (SD)	10.7 (3.0)
BMI-SDS, mean (SD)	3.1 (0.7)
Fat mass percentage, mean (SD)	35.3 (6.2)
Nationality - count (%)	
Danish origin	148 (93%)
Other	11 (6.9%)
Previous unsuccessful weight loss attempt - count (%)	
Yes	66 (42%)
No	91 (58%)
Disposition - overweight - count (%)	
Yes	142 (90%)
No	16 (10%)
Disposition - mental illness - count (%)	
Yes	49 (31%)
No	108 (69%)
Parents relationship - count (%)	
Shared custody	134 (84%)
Not shared custody	25 (16%)

Normally distributed data and categorical variables are reported using mean value with standard deviation (SD) and percentages (%) respectively

### Completers versus non-completers

A total of 64 children left the intervention within the first 1.5 years of the treatment. Of these 59 children (92%) left the intervention at their own initiative (26 reported a reason leaving, 20 did not report a reason, and 13 stopped attending their appointments). Five children (7.8%) left for other reasons (two participated in other interventions, two were stopped by the healthcare professionals (depression and normalization of BMI), and 1 moved away from the involved municipalities). For the 26 children who reported a reason for leaving the intervention, 12 children just wanted to stop, seven children reported a lack of motivation, three children reported logistical problems or that the intervention was too restrictive, two children had a bad experience or bad collaboration with the healthcare professional, and one child stopped due to a competing health issue (autism).

Children who completed the first 1.5 years (completers) of the intervention were found to be younger (10.2 years ± 2.9 vs 11.5 years ± 3.1, p = 0.005) compared to children who left the intervention before the first 1.5 years (non-completers)*.* No difference was observed for BMI-SDS, predispositions for obesity, mental health issues, or psychosocial factors (Table 2).

**Table 2:** Baseline characteristics stratified by duration of treatment (> 1.5 years (completers) and ≤ 1.5 years (non-completers))

Factor	Completers	No completers	*p*-value
*N*	95	64	
Sex - count (%)			0.12
Boys	43 (45%)	37 (58%)	
Girls	52 (55%)	27 (42%)	
Age (years), mean (SD)	10.2 (2.9)	11.5 (3.1)	0.005
BMI-SDS, mean (SD)	3.1 (0.7)	3.2 (0.7)	0.27
Fat mass percentage, mean (SD)	35.1 (5.9)	35.6 (6.7)	0.63
Nationality - count (%)			0.72
Danish origin	89 (94%)	59 (92%)	
Other	6 (6.3%)	5 (7.8%)	
Previous unsuccessful weight loss attempt - count (%)			0.87
Yes	40 (43%)	26 (41%)	
No	54 (57%)	37 (59%)	
Disposition - overweight - count (%)			0.18
Yes	87 (93%)	55 (86%)	
No	7 (7.4%)	9 (14%)	
Disposition - mental illness - count (%)			0.10
Yes	34 (36%)	15 (24%)	
No	60 (64%)	48 (76%)	
Parents relationship - count (%)			0.98
Shared custody	80 (84%)	54 (84%)	
Not shared custody	15 (16%)	10 (16%)	

*p****-***values represent differences between completers and non-completers. Normally distributed data and categorical variables are reported using mean value with standard deviation (SD) and percentages (%) respectively

### Age stratified by tertiles at baseline

The youngest children had a higher BMI-SDS, than both the middle (*p* < 0.001) and the oldest groups of children (*p* = 0.03). In addition, a higher percentage of the youngest children had a nationality other than Danish compared to the middle (*p* = 0.01) and oldest group of children (*p* = 0.047). No other differences were observed between the groups (Table 3).

**Table 3:** Baseline characteristics stratified by age tertiles (i.e., 33.3^rd^ and 66.6^th^) at baseline

Factor	Youngest third	Middle third	Oldest third	*p*-value
*N*	53	53	53	
Sex - count (%)				0.087
Boys	25 (47%)	22 (42%)	33 (62%)	
Girls	28 (53%)	31 (58%)	20 (38%)	
Age (years), mean (SD)	7.4 (1.2)	10.6 (0.7)	14.1 (1.6)	< 0.001
BMI-SDS, mean (SD)	3.4 (0.9)	2.9 (0.6)	3.1 (0.6)	< 0.001
Fat mass percentage, mean (SD)	34.6 (5.9)	34.8 (5.7)	36.4 (6.9)	0.29
treatment_time, mean (SD)	679.0 (334.1)	712.8 (357.6)	488.9 (340.8)	0.002
Nationality - count (%)				0.015
Danish origin	45 (85%)	52 (98%)	51 (96%)	
Other	8 (15%)	1 (1.9%)	2 (3.8%)	
Previous unsuccessful weight loss attempt - count (%)				0.13
Yes	16 (31%)	23 (43%)	27 (51%)	
No	35 (69%)	30 (57%)	26 (49%)	
Disposition - overweight - count (%)				0.92
Yes	48 (91%)	48 (91%)	46 (88%)	
No	5 (9.4%)	5 (9.4%)	6 (12%)	
Disposition - mental illness- count (%)				0.30
Yes	16 (31%)	13 (25%)	20 (38%)	
No	36 (69%)	40 (75%)	32 (62%)	
Parents relationship - count (%)				0.13
Shared custody	43 (81%)	49 (92%)	42 (79%)	
Not shared custody	10 (19%)	4 (7.5%)	11 (21%)	

One-way ANOVA with *p-*values was used to compare the groups. Normally distributed data and categorical variables are reported using mean value with standard deviation (SD) and percentages (%) respectively

The oldest children had a significantly shorter duration of treatment (489 days, SD: ± 341) compared to both the youngest (679 days, SD: ± 334) and the middle group of children (713 days, SD: ± 358). The youngest children were adherent to the interventions for an average of 190 days (95% CI: 60; 320, *p* = 0.005) more than the oldest children, while the middle age group were adherent for an average of 224 days more (95% CI: 89; 358, *p* = 0.00). No difference in duration of the treatment was observed between the youngest and middle group of children (*p* = 0.62) (Table 3).

### Correlation analyses

**Fig. 2:**
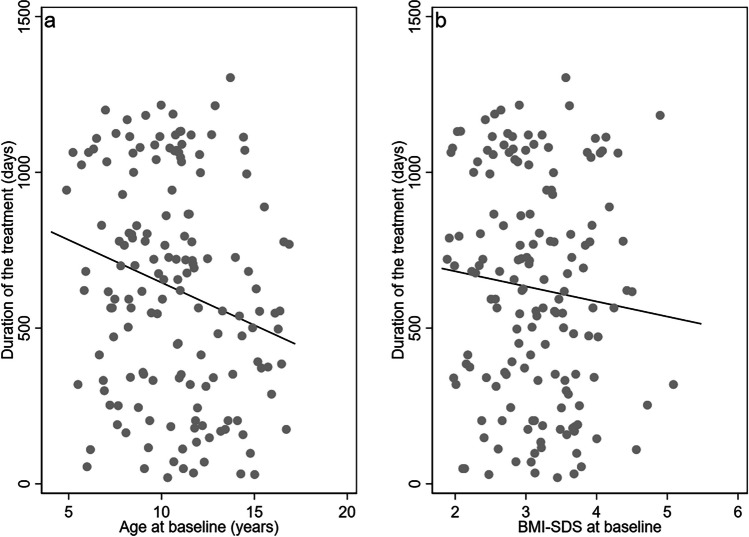
A scatter plot and the unadjusted line of best fit depiction for **a** duration of the treatment as function of age at baseline and **b** duration of treatment as function of BMI-SDS at baseline. All observations in the scatterplots are anonymized and reflects the average of 5 observations

A significant Pearson correlation of −0.23 (*p* = 0.002) between age at baseline and duration of the intervention was observed (Fig. 2a). However, no correlation (*r*: −0.10, *p* = 0.22) (Fig. 2b) was observed between BMI-SDS at baseline and duration of the intervention (Fig. 2b).

### Multiple variable regression analysis

We observed a significant inverse association between age at baseline and duration of the intervention by using an adjusted multivariable regression analysis (−31 days, 95% CI: −50; −13, *p* = 0.001) (Table 4) meaning that an 8-year-old child would leave the intervention 31 days earlier than a 7-year-old child.

**Table 4:** The crude and adjusted association between duration of the intervention (days) and (1) BMI-SDS (SD) and (2) age (years) at baseline, estimated by a multiple variable regression analysis

	Depending variable: duration of the treatment (days)			
Independent variables	Crude		Adjusted	
	β-coefficient (95% CI)	*p*-value	β-coefficient (95% CI)	*p*-value
1) Age at baseline (years)	−27 (−45; −9)	0.003	−31 (−50; −13)	0.001
2) BMI-SDS at baseline (SD)	−49 (−126; 29)	0.22	−76 (−159; 6)	0.07

These models were adjusted for age, sex, and BMI-SDS at baseline

No association (multivariable regression analysis: −76 days, 95% CI: −159; 6, *p* = 0.07) was observed between BMI-SDS at baseline and duration of the intervention (Table 4).

## Discussion

To the best of our knowledge, this is one of the first long-term follow-up studies on the treatment of childhood obesity, demonstrating a dose–response relationship between adherence and age. The present study found a significantly higher age in children with obesity leaving the intervention prematurely, in addition to an inverse association between age at treatment onset and duration of the intervention. These findings suggest that age is an important determinant of adherence to a lifestyle intervention for childhood obesity.

In this study, children in the youngest and middle age-strata were on average adherent to the intervention for 6.3 and 7.4 months longer than participants in the oldest age stratum, respectively. A significant difference was observed in the distribution of children with a nationality other than Danish, but the numbers were very small, and (seen in a Danish setting) without any clinical relevance. A significant difference in anthropometrics was observed when stratifying by age percentiles at baseline. However, fat percentage between the groups was similar so we consider these differences to be related to the age of the participants.

The inverse relationship between age at treatment onset and enrolment time aligns well with previous reports indicating that younger children achieve better weight loss results in lifestyle interventions, compared to older peers [26, 34, 35], and also seem to benefit from the support of their parents especially if the parents engaged in the lifestyle intervention [36]. A reason for this relationship can be that adolescents, due to a higher degree of autonomy, are more likely to drop-out of a lifestyle intervention as compared to younger children [37]. In line with this, it has been reported that better results are achieved if the adolescent and parents have separate consultations [38], which is in contrast to younger children [36]. Adolescents are also reported to be more motivated by peer acceptance [39]. The increased need for independence in adolescents seems to some degree to be opposed to also being more vulnerable and thus susceptible to stress and anxiety [40] and thereby reliant on increased support. In this study, none of the participating children was of legal age. The parents were therefore responsible for the children staying in the intervention. Thus, a lack of motivation from the parents could cause a discontinued treatment regardless of the motivation of the child.

In a qualitative study, Lindelof et al. [41] reported that the communication between adolescents and their parents was important for the outcome of a weight loss intervention and observed that increasing age was inversely related to the involvement and support from the parents. Families who were better at communicating often achieved better results; however, no correction for parental educational level was made.

Age as an important determinant for adherence to lifestyle interventions in childhood obesity has previously been reported [26, 29]. In a cohort study by Zeller et al. [29], early drop-out was associated with age, race, symptom of depression, and family income in an American cohort. However, as opposed to our study, this was a short-term intervention of only 16 weeks duration. In another study, by Danielsson et al. [26], a Swedish cohort of 684 children and adolescents with obesity was treated with a long-term intervention (up to 3 years). The study, only observed a positive association between age at baseline and adherence to the intervention. Citizens in Denmark and Sweden both have access to free healthcare, unlike the American cohort, so it is reasonable to assume that this could be an explanation of the similar findings.

In addition, studies have reported that degree of obesity (i.e., BMI-SDS) could be a determinant for the adherences to a lifestyle intervention program [27, 28]. Denzer et al. described that BMI-SDS at baseline was associated with the total number of visits, concluding that children with higher BMI-SDS were seeking more treatment [27]. Barlow et al. [28] reported that higher BMI was associated with better adherence to an intervention. In the present study, we observed a non-significant inverse relationship between duration of the intervention and BMI-SDS at baseline (*p* = 0.07), suggesting that additional studies are needed in order to clarify the relationship between BMI-SDS and adherence. Parental factors can also be important determinants for the child’s adherence, since several earlier studies have suggested that children from families with low socioeconomic status or obesity were associated with lower adherence to an intervention [31, 30]. However, results from the present study were unable to confirm these previous findings.

These varying results may be explained by the complex interplay of different mechanisms, which may influence a potential weight reduction in children and adolescents. Some of these are related to intrapersonal (i.e., the child’s state of mind) as well as interpersonal (i.e., the child’s relationship with family, friends, and health care professionals) factors that all affect the weight loss process [42, 43].

### Strengths and limitations

One clear strength of this study is that the intervention was constructed as a collaboration between a Department of Paediatrics and a group of specialized community nurses in the municipalities, making the methodology more transferable as it “mimics” real-life conditions. The cohort of children and the treatment included in this study were very homogenous (e.g., small team of healthcare professionals).

A limitation of the study was that only a smaller number of children were included in the intervention, so it is possible that other and weaker associations could be revealed if the study was repeated on a larger scale. Due to the design and the research question, it was not possible to include a control group. However, a control group would have raised the ethical dilemma of not offering treatment (i.e., an intervention) to children living with obesity. Furthermore, the purpose of this study was to identify risk factors for prematurely leaving the intervention, rather than prove the efficacy of the intervention. It was not possible to investigate possible associations between adherence to the interventions and lifestyle factors at baseline (i.e., physical activity and dietary habits)*,* because data were not collected. Another limitation is the broad age range among the included children, which complicates the comparison of BMI-SDS, BMI, fat percentage, etc., due to the pubertal growth.

## Conclusion

This study reports a robust inverse association between age at treatment onset and the duration of the intervention for children with obesity. The study emphasizes the importance of initiating treatment of children living with obesity as early as possible. Another important result and in consensus with current knowledge is that one intervention does not fit all children. We believe that future research involving children and adolescents living with obesity should focus on intrapersonal characteristics such as motivation, autonomy, and vulnerability, as these may influence the outcomes of weight loss interventions.

## Data Availability

Data and materials will be available and shared on reasonable request to the corresponding author.

## Abbreviations

BMI-SDS: Body Mass Index Standard Deviation Score
CI: Confidence intervals
IQR: Interquartile range
SD: Standard deviations
TCOCT: The children’s obesity clinics treatment
